# Impacts of gender and age on meibomian gland in aged people using artificial intelligence

**DOI:** 10.3389/fcell.2023.1199440

**Published:** 2023-06-15

**Authors:** Binge Huang, Fangrong Fei, Han Wen, Ye Zhu, Zhenzhen Wang, Shuwen Zhang, Liang Hu, Wei Chen, Qinxiang Zheng

**Affiliations:** ^1^ School of Ophthalmology and Optometry, Eye Hospital, Wenzhou Medical University, Wenzhou, China; ^2^ Zhejiang Provincial Center for Disease Control and Prevention, Hangzhou, China

**Keywords:** meibomian glands, meibomian gland dysfunction, meibography, artificial intelligence, aging

## Abstract

**Purpose:** To evaluate the effects of age and gender on meibomian gland (MG) parameters and the associations among MG parameters in aged people using a deep-learning based artificial intelligence (AI).

**Methods:** A total of 119 subjects aged ≥60 were enrolled. Subjects completed an ocular surface disease index (OSDI) questionnaire, received ocular surface examinations including Meibography images captured by Keratograph 5M, diagnosis of meibomian gland dysfunction (MGD) and assessment of lid margin and meibum. Images were analyzed using an AI system to evaluate the MG area, density, number, height, width and tortuosity.

**Results:** The mean age of the subjects was 71.61 ± 7.36 years. The prevalence of severe MGD and meibomian gland loss (MGL) increased with age, as well as the lid margin abnormities. Gender differences of MG morphological parameters were most significant in subjects less than 70 years old. The MG morphological parameters detected by AI system had strong relationship with the traditional manual evaluation of MGL and lid margin parameters. Lid margin abnormities were significantly correlated with MG height and MGL. OSDI was related to MGL, MG area, MG height, plugging and lipid extrusion test (LET). Male subjects, especially the ones who smoke or drink, had severe lid margin abnormities, and significantly decreased MG number, height, and area than the females.

**Conclusion:** The AI system is a reliable and high-efficient method for evaluating MG morphology and function. MG morphological abnormities developed with age and were worse in the aging males, and smoking and drinking were risk factors.

## 1 Introduction

Meibomian gland dysfunction (MGD) is a chronic and diffuse disease in the meibomian glands (MG), which is the major type of evaporative dry eye disease (DED) and commonly seen in eye clinic. It is characterized by terminal duct obstruction with or without the abnormity of the glandular secretion, and usually accompanied by different levels of meibomian gland loss (MGL) (Nelson et al., 2011). Among the diverse intrinsic and external factors contributing to MGD, aging is a major one due to the development of meibomian glands atrophy with structural and functional abnormities (Schaumberg et al., 2011; Yeotikar et al., 2016; Arita et al., 2017). Furthermore, the sex hormone receptor has been found in ocular surface, through which sex hormone regulates metabolism, gene expression and tear secretion (Schirra et al., 2005; Suzuki et al., 2008; Versura et al., 2015). Some population-based studies have found that the prevalence of MGD in males is higher than that in females at any age (Viso et al., 2011; Siak et al., 2012; Hashemi et al., 2017; Hashemi et al., 2021). And abnormal lid margin and MG morphology are more common in aging males (Den et al., 2006). The effect of hormones on the ocular surface is still controversial, since the deficiency of estrogen promotes the occurrence of dry eye disease in females (Grasso et al., 2021; Hat et al., 2023). To analyze the influence of gender on the MGs, we evaluated the MG morphology and MGL level of different genders in the current study, and the risk factors of smoking and drinking were also taken into consideration.

At present, there has been many studies on MGD in the elderly, but few on the changes of MG parameters. Nowadays the clinical diagnosis of MGD still lacks objective evaluations, and depends on the subjective judgment. Recently, the technology of meibography has been constantly developed, and is able to provide objective means to evaluate MG status using artificial intelligence (AI) (Adil et al., 2019; Deng et al., 2021; Xiao et al., 2021; Liu et al., 2022; Zhang et al., 2022). In order to obtain objective results, we evaluated the relationship of MG parameters identified by AI with age and gender in the current study. We enrolled the aged people on a hospital-based group. After collecting meibography and eye surface examination, the deep learning model developed in our early study (Liu et al., 2022; Zhang et al., 2022) was used to identify gland parameters, including gland area, area density, number, height, width and tortuosity. The associations between AI-reported MG parameters and traditional values of MGL, lid margin abnormities were assessed, as well as the age and gender effects on MGD.

## 2 Materials and methods

### 2.1 Subjects

In this prospective cross-sectional study, a total of 119 subjects aged at ≥ 60 years were recruited from the outpatient department of the Eye Hospital of Wenzhou Medical University between September 2020 and May 2021. The exclusion criteria included: ocular or systemic diseases associated with dry eye disease such as Sjögren’s syndrome, graft *versus* host disease, collagen angiopathy, except MGD; any active eye disease such as infection and acute glaucoma; a previous history of ophthalmologic surgery; structure abnormity of the eyelid, conjunctiva and cornea; history of contact lens wear within 6 months; history of systemic or ocular medication treatment within 6 months such as hormones, antiallergic drugs, immunosuppressants, except artificial tears without preservatives. The whole procedure of the study was approved by the Institutional Review Board of Wenzhou Medical University and adhered to the tenets of the Declaration of Helsinki (No. 2020-096-K-83), and the written informed consent was obtained from all subjects before participating in the study.

All the subjects were examined by Binge Huang. Only the right eyes were evaluated. The examinations were conducted in sequence: 1) completing an ocular surface disease index (OSDI) questionnaire; 2) noninvasive meibography by Keratograph 5M (K5M; Oculus Optikgeräte GmbH, Wetzlar, Germany); 3) slit-lamp biomicroscopy including diagnosis and staging of MGD and assessment of lid margin and meibum.

### 2.2 Diagnosis and staging of MGD

The subjects were diagnosed with normal, asymptomatic MGD and MGD based on the “Expert consensus of diagnosis and treatment of meibomian gland dysfunction in China (2017)” (China branch of Asian dry eye Association et al., 2017), in which subjects with asymptomatic MGD were not diagnosed as MGD. Patients with MGD were divided into 3 stages according to condition of lid margin, meibomian gland orifices and meibum based on the clinical judgment of the same ophthalmologist.

### 2.3 Meibography collection and MG parameters detection

Meibography images of the upper and lower lids were conducted by the noncontact infrared camera system in the K5M. The MGL score was graded according to the meibography results as 0 (no loss of meibomian glands), 1 (area loss was less than one third of the total meibomian gland area), 2 (area loss was between one third and two thirds), and 3 (area loss was more than two thirds). Both of the upper and lower lid were examined and the total summing score was used for analysis. And the participants were then divided into two groups depending on the score of MGL: the low MGL group (LMGL) with meiboscore <3, and the high MGL group (HMGL) with meiboscore ≥3. Images of upper lid were analyzed based on a novel MG morphology analytic system we developed recently (Zhang et al., 2022). This AI system automatically segmented MGs and quantitatively analyzed the MGs’ morphological features (gland area, density, number, height, width and tortuosity).

### 2.4 Lid margin and meibum assessment

The lid margin and meibum examinations were conducted according to the Arita R et al.'s grading methods (Arita et al., 2016). Lid margin telangiectasia was graded as 0 (no sign of telangiectasia), 1 (mild sign), 2 (moderate sign affecting <1/2 of the lid margin) and 3 (severe sign affecting ≥1/2 of the lid margin). Lid margin irregularity on the mucocutaneous junction was graded as 0 (marx line (ML) does not touch the meibomian orifice (MO)), 1 (parts of ML touch MOs), 2 (ML crosses MOs), 3 (ML touches the lid margin side of MOs). Lid margin thickness was assessed as 0 (no thickening), 1 (mild thickening) and 2 (severe thickening). MO plugging was graded as 0 (no sign of plugging), 1 (mild covering on the MOs), 2 (moderate plugging and hunch), 3 (severe plugging or atrophy). Lipid extrusion test (LET) was used to evaluate the degree of ease with which meibum could be expressed and the scores of LET at the central area of both upper and lower lid were added together: grade 0, clear meibum readily expressed; grade 1, cloudy meibum expressed with mild pressure; grade 2, cloudy meibum expressed with more than moderate pressure; 3, meibum could not be expressed even with strong pressure (Shimazaki et al., 1998). The meibum quality from the 8 MOs at the central area of the lower lid was assessed: grade 0, clear meibum expressed with digital pressure; grade 1, cloudy meibum expressed; grade 2, cloudy meibum expressed with granules; 3, thick meibum expressed. The scores of the 8 MOs were summed for analysis.

### 2.5 Statistical analysis

Statistical analysis was performed using the SPSS statistics 26.0 (IBM corp., Armonk, NY, USA). Values were expressed as the mean ± standard deviation (SD) or range or median (interquartile range [IQR]). Normal distribution of data was tested using Shapiro Wilk test. Independent sample *t*-test and one-way ANOVA with LSD correction were used for comparison when the variance was homogeneous, or otherwise the Mann-Whitney U rank test. Spearman correlation analysis was conducted to evaluate the strengths of association between the parameters. Differences in prevalence among categorical variables were compared using the Chi Square test. Two-tailed *p* < 0.05 was considered as a statistically significant difference.

## 3 Results

**FIGURE 1:**
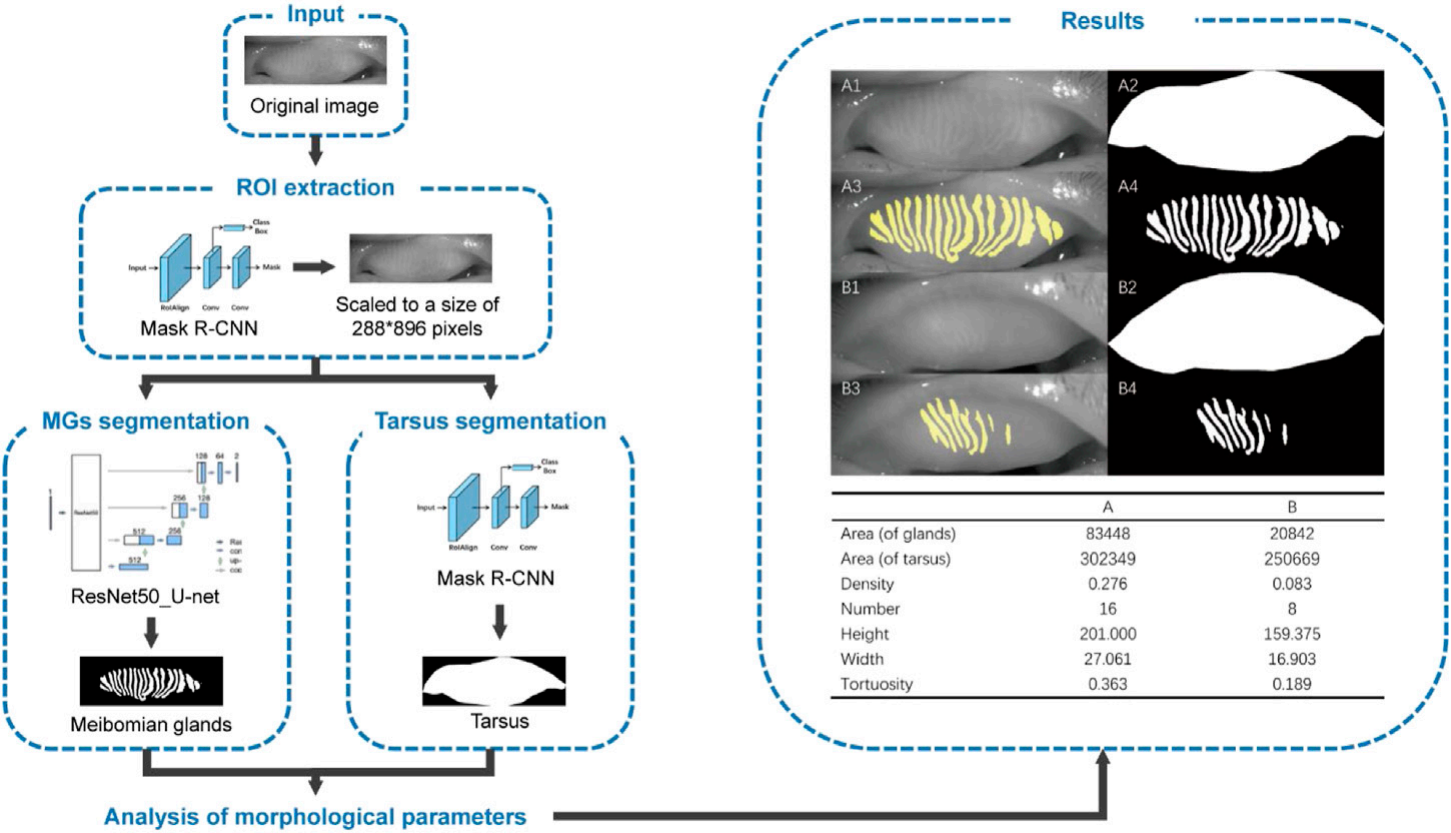
Overall pipeline of the deep learning-based artificial intelligence for predicting morphological parameters from K5M images. (A1) an original meibography image of the upper lid from a normal subject, (B1) an original meibography image of the upper lid from a patient with HMGL, (A2/B2) the AI predicted boundaries of the tarsus, (A3/A4/B3/B4) the AI predicted segmentation MGs. Notes: ROI, region of interest; MG, meibomian gland.

A total of 119 aging subjects (119 eyes; 46 males, 73 females) were identified. The mean age of the participants was 71.61 ± 7.36 (mean ± standard deviation) years (range: 60-89 years). 95.8% subjects were initially diagnosed with dry eye syndrome based on the OSDI score (26.98 ± 10.27 points) (Grubbs et al., 2014). There was no significant difference between genders (male: 26.780 ± 12.220, female: 27.110 ± 8.905, *p* = 0.873) in OSDI score. Figure 1 shows the workflow of the deep learning model for predicting morphological parameters from K5M images and provides two typical cases of predicted meibomian gland segmentation and parameters estimation for the upper lids. The tarsus segmentation model was based on Mask R-CNN (He et al., 2020). The ResNet50_U-net was reported previously (Zhang et al., 2022).

### 3.1 The associations between MG parameters

**FIGURE 2:**
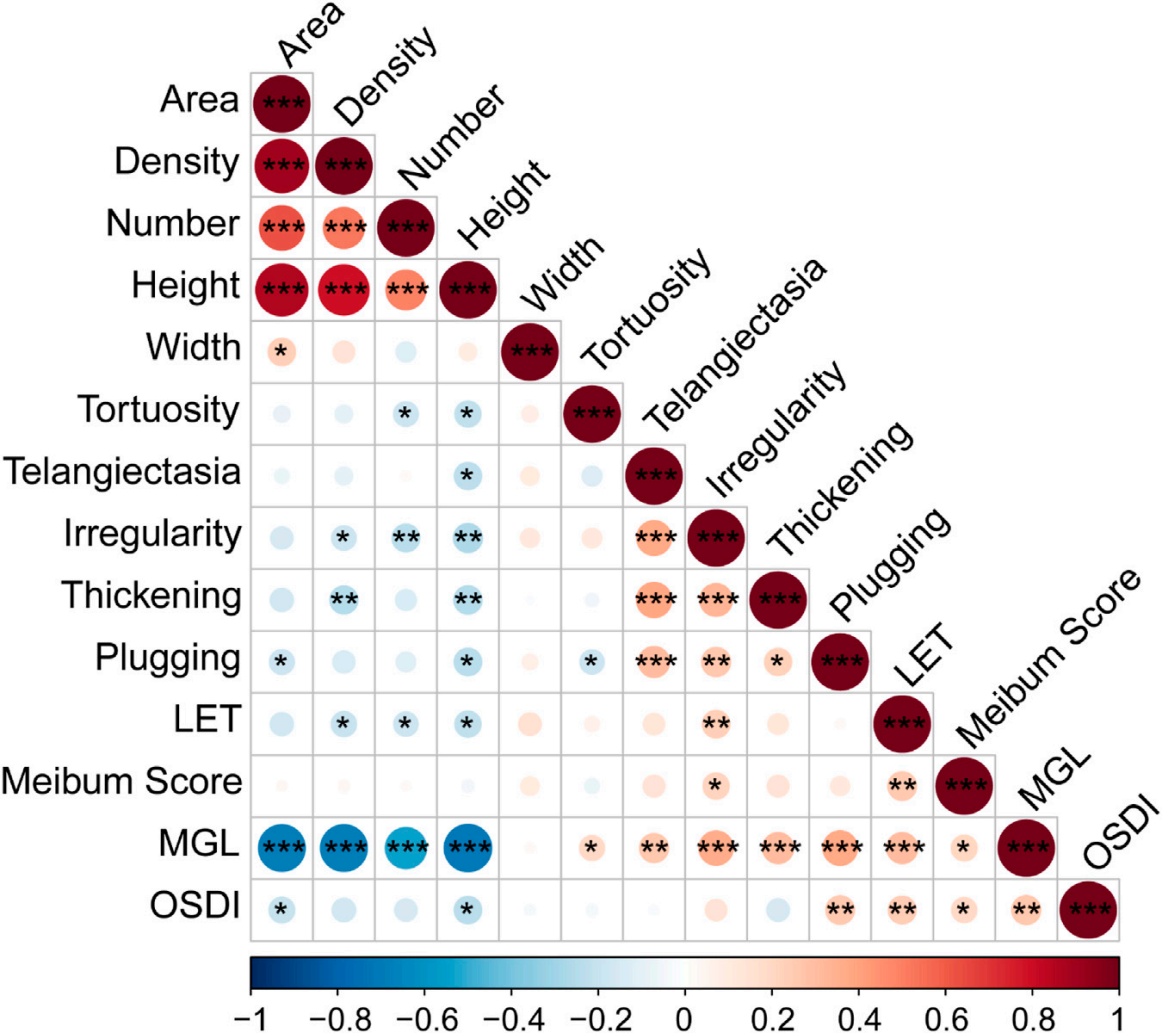
Spearman Correlation of MG parameters and OSDI. Notes: MG, meibomian gland; LET, lipid extrusion test; MGL, meibomian gland loss; OSDI, Ocular Surface Disease Index questionnaire. * *p* < 0.05; ** *p* < 0.01; *** *p* < 0.001.

The correlations between the AI reported meibomian gland morphology parameters (MG area, density, number, height, width and tortuosity), lid margin parameters (telangiectasia, irregularity, thickening, plugging and LET), meibum score, MGL and OSDI score were evaluated (Figure 2). Among the MG morphology parameters, the MG height showed the strongest correlation with all the lid margin parameters (r < −0.212, *p* < 0.020); MG area was relevant with plugging (r = −0.19, *p* = 0.038); MG density was relevant with irregularity (r = −0.185, *p* = 0.044), thickening (r = −0.165, *p* = 0.008) and LET (r = −0.164 *p* = 0.032). OSDI score had relations with MGL (r = 0.261, *p* = 0.004), MG area (r = −0.205, *p* = 0.025), MG height (r = −0.233, *p* = 0.011), plugging (r = 0.255, *p* = 0.005) and LET (r = 0.240, *p* = 0.009), but not significant with MG density (r = −0.170, *p* = 0.064). Furthermore, we found MGL were highly correlated with MG area (r = −0.686, *p* < 0.001), density (r = −0.689, *p* < 0.001), number (r = −0.531, *p* < 0.001), height (r = −0.707, *p* < 0.001), tortuosity (r = 0.193, *p* = 0.036), telangiectasia (r = 0.262, *p* = 0.004), irregularity (r = 0.368, *p* < 0.001), thickening (r = 0.301, *p* < 0.001), plugging (r = 0.374, *p* < 0.001), LET (r = 0.300, *p* < 0.001), meibum score (r = 0.208, *p* = 0.023). Thus, AI system is able to output reliable MG morphology parameters, which have strong association with the traditional manual evaluation of MGL and lid margin parameters but are more efficient and objective.

### 3.2 Age was a risk factor for MGD and MGL

Among the meibomian gland parameters, the number of MGs degenerated with age significantly, which decreased from 15.62 ± 3.99 in subjects aged 60-69 years to 12.12 ± 5.56 in those aged 80-89 years (r = −0.208, *p* = 0.023) (Table 1). And the MGL level and parameters of the lid margin including the telangiectasia, irregularity, thickening and plugging, also aggravated with age with significant correlations (r ≥ 0.229, *p* ≤ 0.012) (Table 1).

**TABLE 1:** Spearman Correlation of MG parameters with age (mean ± standard deviation).

	60-69 (N = 52)	70-79 (N = 50)	80-89 (N = 17)	R	P
Morphological parameters					
Area	44580.596 ± 20039.422	43229.740 ± 22284.460	33976.235 ± 21592.014	−0.100	0.280
Density	0.154 ± 0.065	0.140 ± 0.068	0.127 ± 0.073	−0.102	0.269
Number	15.620 ± 3.986	14.880 ± 5.583	12.120 ± 5.555	−0.208	0.023
Height	134.103 ± 36.834	131.940 ± 45.13	116.180 ± 39.801	−0.113	0.220
Width	20.072 ± 3.899	20.897 ± 3.822	21.446 ± 4.360	0.145	0.116
Tortuosity	0.312 ± 0.071	0.321 ± 0.166	0.280 ± 0.070	−0.078	0.396
Lid margin abnormality parameters					
Telangiectasia	0.870 ± 0.627	1.460 ± 0.908	1.410 ± 0.795	0.229	0.012
Irregularity	0.730 ± 1.012	1.180 ± 0.873	1.290 ± 1.105	0.341	<0.001
Thickening	0.250 ± 0.519	0.600 ± 0.670	0.470 ± 0.514	0.266	0.003
Plugging	0.750 ± 0.837	1.280 ± 1.031	1.650 ± 1.115	0.291	0.001
LET	1.400 ± 1.287	1.500 ± 1.298	1.060 ± 1.435	−0.020	0.831
Others					
Meibum Score	9.310 ± 5.147	9.420 ± 5.897	11.880 ± 5.611	0.114	0.215
MGL	2.810 ± 1.522	3.380 ± 1.772	3.530 ± 1.700	0.235	0.010

Notes: MG, meibomian gland; LET, lipid extrusion test; MGL, meibomian gland loss.

**FIGURE 3:**
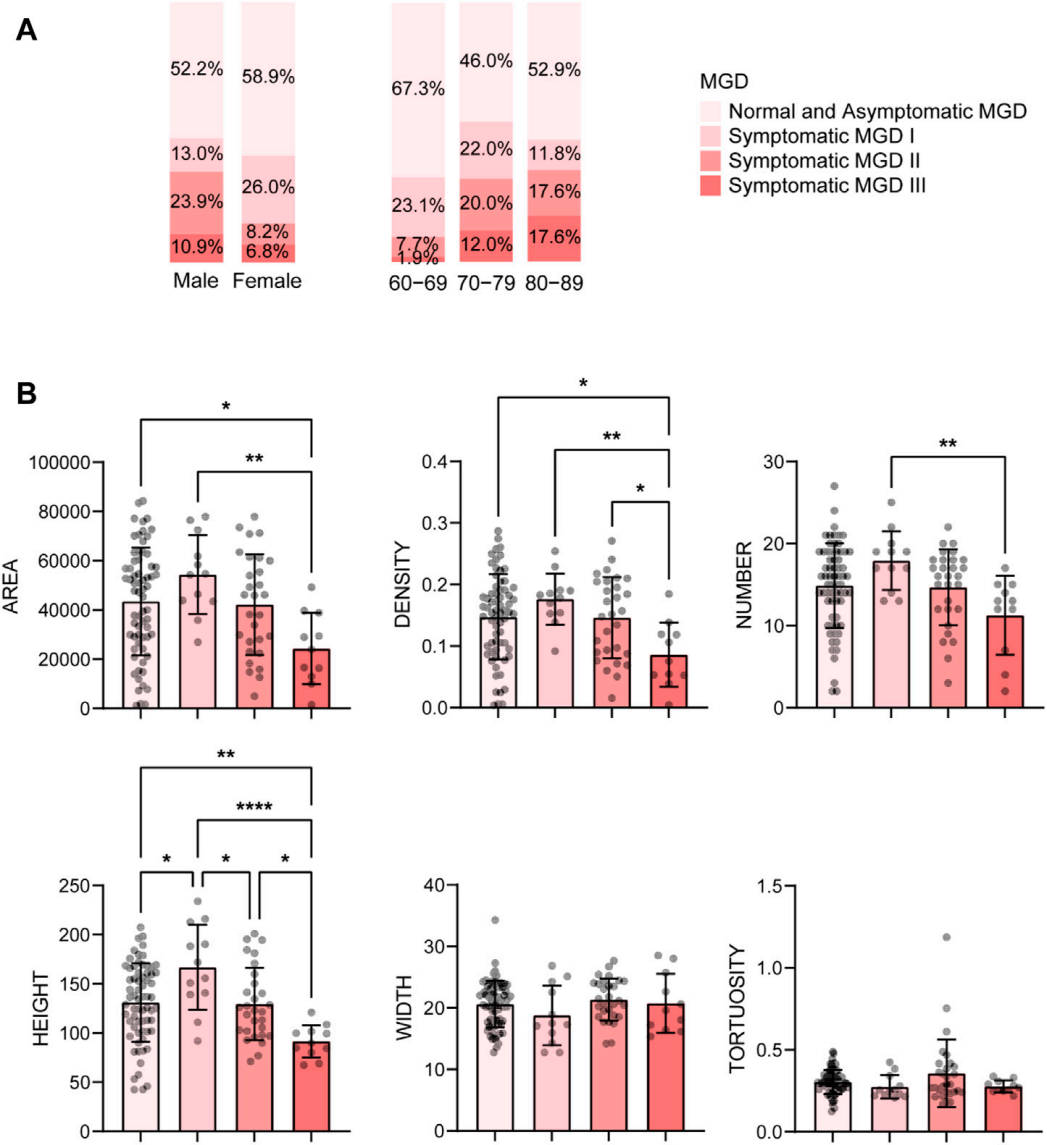
Percent of different severity of MGD in different gender and age groups **(A)** and mean ± standard deviation of MG morphological parameters in each severity group **(B)**. Notes: MGD, meibomian gland dysfunction. * *p* < 0.05; ** *p* < 0.01; *** *p* < 0.001; **** *p* < 0.0001.

**FIGURE 4:**
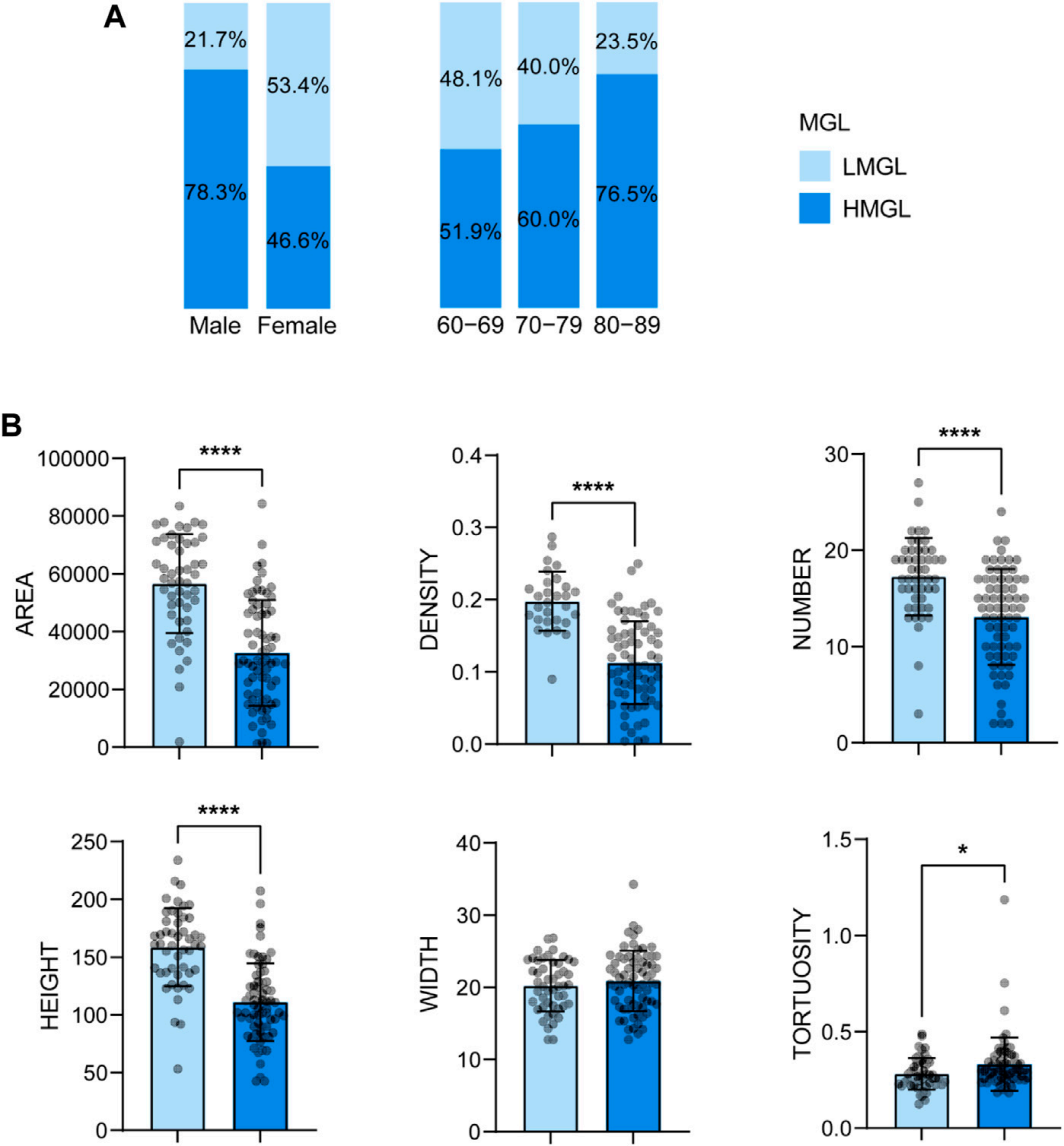
Percent of HMGL and HMGL in different gender and age groups **(A)** and mean ± standard deviation of MG morphological parameters in each severity group **(B)**. Notes: MGL, meibomian gland loss; LMGL, low MGL; HMGL, high MGL. * *p* < 0.05; ** *p* < 0.01; *** *p* < 0.001; **** *p* < 0.0001.

With age grows, the severity of MGD increases. The percentage of severe MGD (level Ⅱ and Ⅲ) was highest in subjects aged 80-89 years (9.6% in 60-69, 32.0% in 70-79, 35.2% in 80-89, see Figure 3A), and the MG parameters including gland area, density, number and height, decreased with the MGD level increased (Figure 3B). Besides, there was a strong correlation between MGD severity and age (r = 0.350, *p* < 0.001), and the proportion of HMGL subjects also increased with age (Figure 4A). The mean age of patients with LMGL was 69.49 ± 6.61, and the mean age of patients with HMGL was 73.1 ± 7.54 (*p* = 0.008). And the gland area, density, number and height, were much lower in HMGL subjects than the LMGL group (*p* < 0.001) (Figure 4B).

### 3.3 MG morphology differs in aging males and females

**FIGURE 5:**
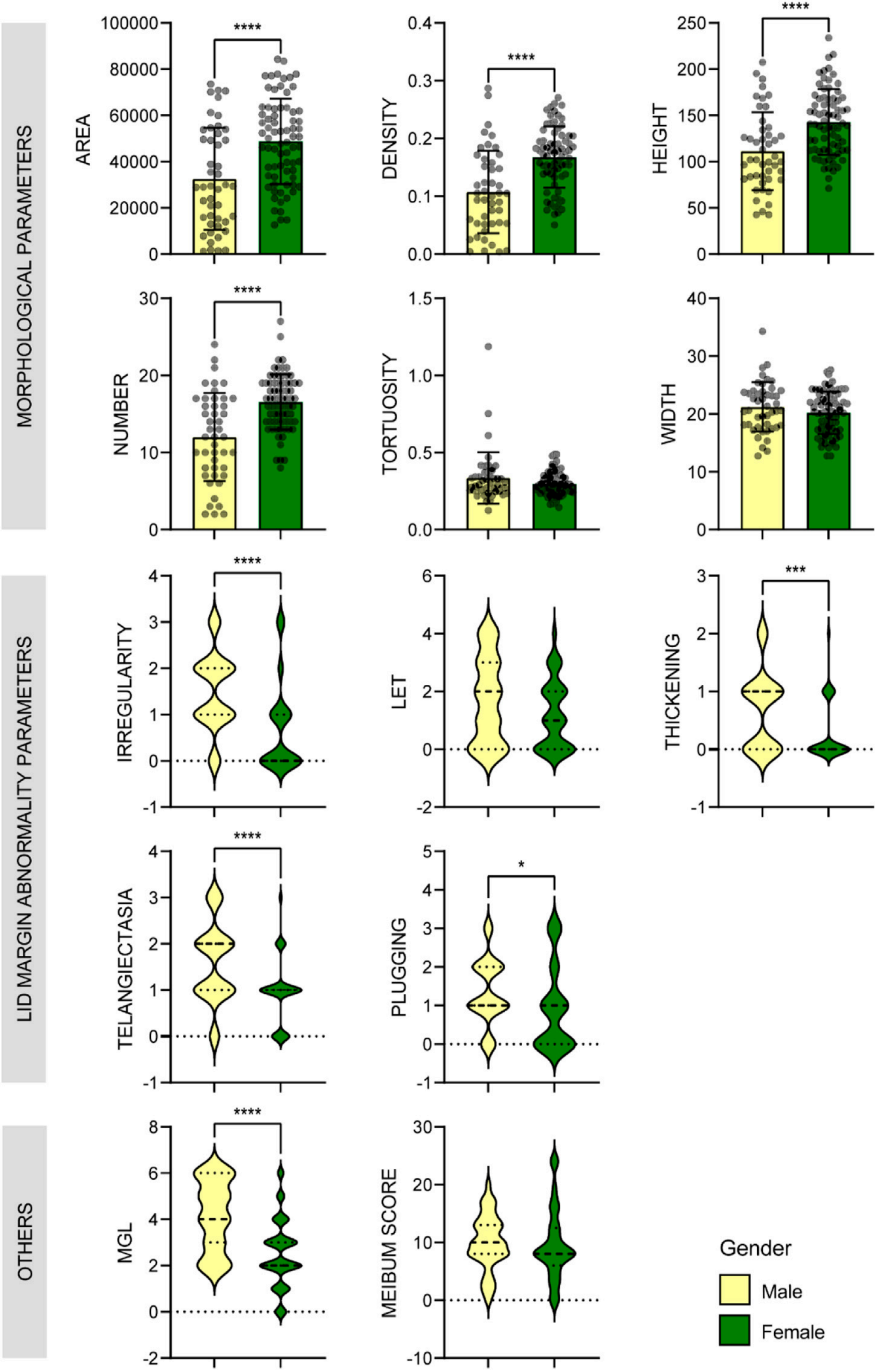
Comparison of MG parameters between male and female groups. Bar plots with mean ± standard deviation were used to show scale variables and violin plots were used to show ordinal variables. * *p* < 0.05; ** *p* < 0.01; *** *p* < 0.001.

AI found that there were significant differences in MG number, height, and area between the aging males and females, and the values were all much lower in the male group (*p* < 0.001) (Figure 5). Correspondingly, the MGL and lid margin parameters including telangiectasia, irregularity, thickening, plugging, were also severely higher in the males (*p* ≤ 0.034) (Figure 5). Among the 46 male subjects, 22 (47.8%) were diagnosed with MGD, of which 34.8% were moderate or severe; of the 73 female subjects, 30 (41.1%) were MGD, in which 15.0% were moderate or severe (Figure 3A). The severity of male MGD was significantly higher than that of female (Chi square test: χ^2^ = 7.899, *p* = 0.048). These results demonstrated that the MG morphology and function were significantly worse in aging males compared with the females. And with the severity of MGD increases, the AI reported values of MG area, density, number and height decreases significantly (*p* < 0.05).

**FIGURE 6:**
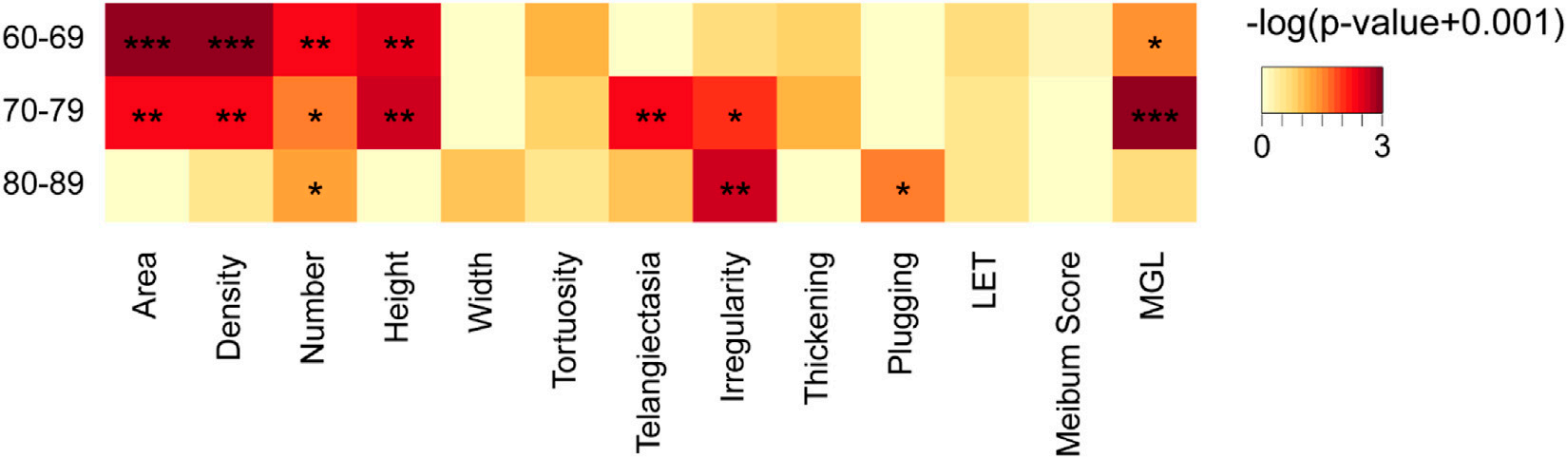
Comparison of meibomian gland and ocular surface parameters of males and females in different age groups. * *p* < 0.05; ** *p* < 0.01; *** *p* < 0.001.

Besides, the ratio of HMGL subjects was much higher in males (36/46, 78.26%) than that in the females (34/73, 46.6%) (Chi square test: χ^2^ = 11.696, *p* = 0.001) (Figure 4A). And in different age groups, the differences in MG morphology, lid margin parameters and MGL level were significant between the males and females, and they were the most marvelous in the 70-79 years age groups (Figure 6).

**FIGURE 7:**
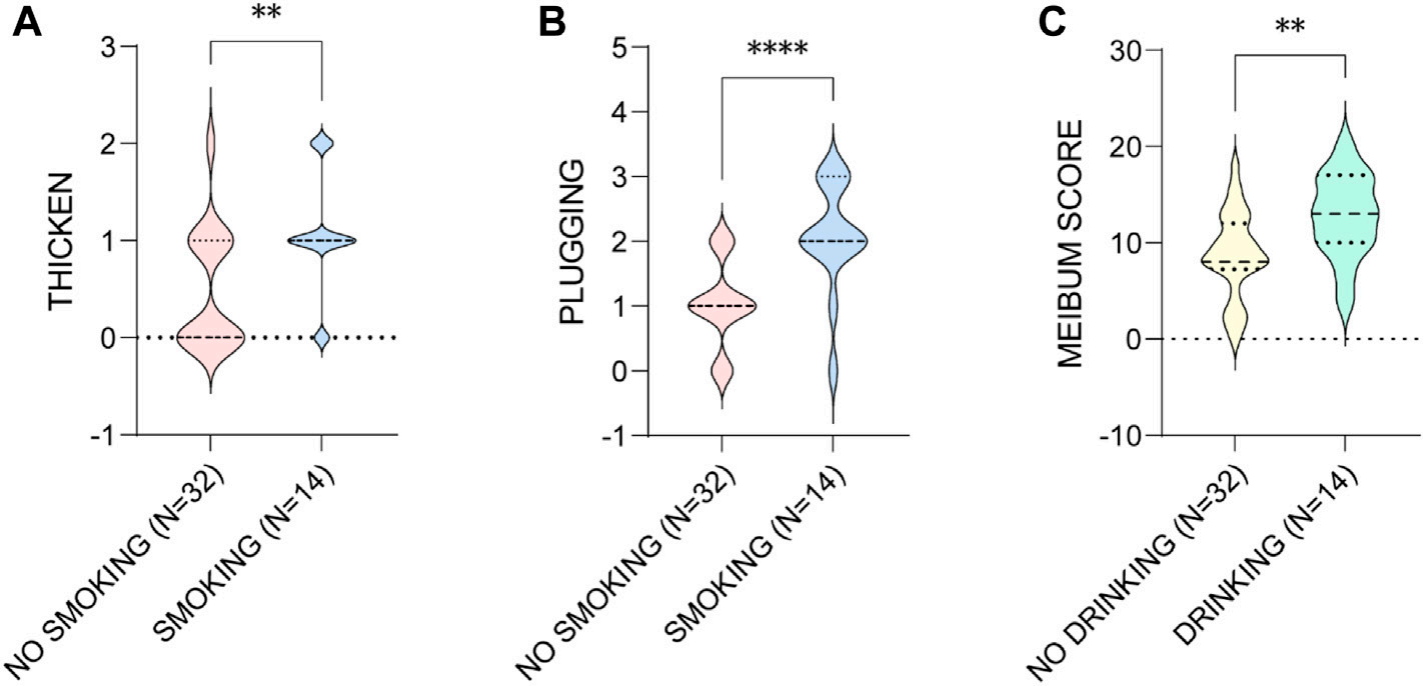
The influence of smoking on the **(A)** thicken of MGs and **(B)** the plugging of MOs, and **(C)** drinking on the meibum score in aging males. * *p* < 0.05; ** *p* < 0.01; *** *p* < 0.001; **** *p* < 0.0001.

Then we found that smoking and drinking were both risk factors associated with the worse MG presentations in the males. The aging males who smoke showed significant higher levels of lid margin thickening (*p* = 0.006) and plugging (*p* < 0.001), and the ones who drinks presented greater meibum score (*p* = 0.006) (Figure 7).

## 4 Discussion

Although many standardized grading scales have been developed to assess the morphology severity of MGs at present (Arita et al., 2009; Daniel et al., 2019; Wang et al., 2022), the scores are based on the subjective judgment of the examiner. AI has been found of excellent accuracy, efficiency and consistency to evaluate MG parameters, helping to diagnosis and even preclinical diagnosis of MGD (Fasanella et al., 2016; Xiao et al., 2021; Zhang et al., 2021). The current study explored the influence of age and gender on MG morphology and correlations among MG parameters in the elderly based on an AI system.

Recently, the development and combination of meibography and AI technology have provided the possibility for objective and efficient identification of MG parameters (Deng et al., 2021). K5M is a non-invasive infrared meibography used routinely in clinical to evaluate morphology of MGs. It could generate the MG results in 1 min without any discomfort. Koh et al. (Koh et al., 2012) firstly reported an algorithm to output MG results from K5M images in 2012, incorporating a deep learning model to differentiate healthy and unhealthy MGs to help diagnoses of MGD. Nowadays AI has been consistently developed and is able to detect various MG parameters and minimize the influence of artifacts (Maruoka et al., 2020; Deng et al., 2021). In the current study, we applied a deep learning model, which was reported previously (Liu et al., 2022; Zhang et al., 2022) to segment MGs from images and compute MG parameters. The proposed MG density can diagnose MGD with high sensitivity and specificity. The results show that the area, density, number and height of MGs present strong negative correlation coefficient with the subjective parameter of MGL, indicating that the AI generated MG parameters are reliable consistent with the traditional evaluation of MGL. In addition, the values of MG area, density, number and height were uniformly associated with the severity of MGD. However, there was a discrepancy in our results that the MG morphological parameters of normal and asymptomatic MGD group were lower than those of mild MGD group. It was possible that some patients with significant abnormal morphology of MG or MGL, may report no symptoms and were diagnosed as non-MGD.

The decreases in MG height, width and number lead to MGL, and lid margin abnormalities are also associated with MGL development (Arita et al., 2008; Ha et al., 2021). Our results found that, lid margin telangiectasia, irregularity, thickening, plugging and LET were significantly correlated with MG height and MGL level but not MG width or number, demonstrating that the MG height might be more sensitive to reflect MGL in patients with abnormal lid margin. In addition, we found MG density may have higher correlation with the change of lid margin parameters than MG area. These results indicate that the MG height and density have more clinical significance than other parameters, and may be helpful for the diagnosis and severity assessment of MGD.

The OSDI is a 12-item questionnaire designed to assess ocular symptoms related to dry eye disease and their effect on vision function. Our results found that OSDI was associated to MGL, MG area, MG height, lid margin plugging and LET. However, it was of no difference between the males and females, but the MG parameters and the severity of MGD and MGL were significantly different between them. The reasons for these discrepancies are unclear. Daniel et al. (Daniel et al., 2019, Daniel et al., 2020) found no morphological features of MG related to OSDI in patients with moderate to severe dry eye disease. Adil et al. (Adil et al., 2019) also found OSDI did not correlate with any MG morphologic parameter in MGD patients, however the OSDI at different meibogrades had statistical differences. Their results were not contradictory to ours on the base of different design and subjects.

Sex hormones modulate gene expression in MGs and play an important role in ocular surface health (Schirra et al., 2005; Suzuki et al., 2008). Epidemiological data shows the prevalence rates of MGD ranging from 39% to 68% in Asia, and mainly in the elderly and male (Siak et al., 2012; Alghamdi et al., 2016; Hashemi et al., 2017). In the current study, the prevalence of symptomatic MGD and HMGL in aging males were much higher than that in the females. And most of the MG parameters of aging males were worse than those of the females. Studies have shown that androgens promote meibum secretion, which leads to higher incidence of dry eye disease in women and obstructive MGD in older men (Schirra et al., 2005). The effect of estrogen on the ocular surface is still controversial. It is generally accepted that excessive exposure and deficiency of estrogen promote the occurrence of dry eye disease (Schirra et al., 2009; Versura et al., 2015). So we speculated that the habits of smoking and drinking may play a more important role on MGD and MGL development, since the subjects who smoke in the male group had significant higher levels of lid margin thickening and plugging, and the ones who drinks had greater meibum score. None of the females in the current study had the habit of smoking or drinking. Similarly, it was reported that smoking index was significantly correlated with the scores of lid margin abnormality and meibum (Wang et al., 2016), and smoking is associated with dry eye and MGD (Carreira et al., 2022).

Several previous studies have reported the prevalence of MGD increases with age (Siak et al., 2012; Alghamdi et al., 2016; Hashemi et al., 2017), which was consistent with our results. And in the aging people over 60 years old, MG function became severely worse with age climbs, especially for the lid margin abnormities, MGL level and MG number, which was in accordance with previous reports (Arita et al., 2008; Ban et al., 2013). In addition, the sample size of the current study could be enlarged in the following investigations to exert stronger conclusions, and we aim to improve the AI system to reduce the impact of artifacts and exploring more MG parameters of clinical value in the next step.

Collectively, the AI system is a reliable and fast method for evaluating MG parameters. Using this method, we found gender and age influenced various MG parameters, and were risk factors for the health of MGs in the elderly.

## Data Availability

The raw data supporting the conclusions of this article will be made available by the authors, without undue reservation.
